# Enhancement of Dye-Sensitized Solar Cells Efficiency Using Mixed-Phase TiO_2_ Nanoparticles as Photoanode

**DOI:** 10.1155/2017/9152973

**Published:** 2017-08-15

**Authors:** Yi-Hua Fan, Ching-Yuan Ho, Yaw-Jen Chang

**Affiliations:** Department of Mechanical Engineering, Chung Yuan Christian University, Chung-Li, Taiwan

## Abstract

Dye-sensitized solar cell (DSSC) is a potential candidate to replace conventional silicon-based solar cells because of high efficiency, cheap cost, and lower energy consumption in comparison with silicon chip manufacture. In this report, mixed-phase (anatase and rutile nanoparticles) TiO_2_ photoanode was synthesized to investigate material characteristics, carriers transport, and photovoltaic performance for future DSSC application. Field-emission scanning electron microscope (SEM), X-ray diffraction (XRD), photoluminescence (PL), and UV-visible spectroscopy were used to characterize mixed TiO_2_ particles. Subsequently, various mixed-phase TiO_2_ anodes in DSSC devices were measured by electrical impedance spectra (EIS) and energy efficiency conversion. The overall energy conversion efficiency of DSSC chip was improved as a result of the increase of rutile phase of TiO_2_ (14%) in anatase matrix. Synergistic effects including TiO_2_ crystallization, reduction of defect density level in energy band, longer lifetime of photoexcited electrons, and lower resistance of electron pathway all contributed to high efficiency of light energy conversion.

## 1. Introduction

Significant demand for energy gives rise to the depletion of fossil resources, global warming, and climate change; thus energy substitutes are always urgent mission to protect living condition without losing life convenience and economic benefits. Among all alternatives, dye-sensitized solar cells (DSSCs) with simple structure and acceptable conversion of sunlight into electricity with low cost and high efficiency have attracted much attention. Traditionally, TiO_2_ semiconductor acting as anode electrode is attached to sensitizer ruthenium dye and is involved in volatile liquid electrolyte containing I^−^/I^3^-redox couple and counterelectrode to form a complete DSSC cell [1–3]. Therefore, the DSSC performance strongly depends on the TiO_2_ characteristics such as its morphology, phase compositions, and other properties [4, 5]. Recently, many studies have offered one-dimensional TiO_2_ nanomaterials such as nanowires and nanotubes which offer high surface-to-volume ratio in the structure of TiO_2_ photoanode because abundant dye can be loaded on the TiO_2_ photoanode surface to maximize the amount of photogeneration [6]. In efficient DSSC operation, one cycle of photon-to-electricity conversion is completed by fast electron injection from a photoexcited dye into the TiO_2_ conduction band and subsequently dye regeneration and holes transportation to the counterelectrode. Lower resistance of charge pathways and electron-hole recombination rate of photogenerated carriers are necessary for longer electron diffusion length and extending lifetime in TiO_2_ photoanode and to obtain good collection efficiency [7, 8]. Therefore, the TiO_2_ semiconductor with adequate band gap and pathway resistance of electron transport plays an important role in obtaining the most efficient DSSCs. Although 1D of nanocrystalline TiO_2_ is preferred to act as photoanode by proposing enough dye attaching, well order and good arrangement patterns are difficult to fabrication using low cost process. Besides, minimized interfacial charge recombination has to be considered during carrier transport and final conversion efficiency. To decrease interfacial recombination and increasing electron lifetime, metal oxides of core shell 1D configuration on top of the transparent TiO_2_ film have been investigated using ZrO_2_ and ZnO. In order to adjust conduction band position, overcoat materials Mg(OH)2, Zn(OH)2 proposed more negative conduction band in comparison with TiO_2_; thus these metal hydroxides were applied as a blocking TiO_2_ layer at the FTO/TiO_2_ interface to decrease electron leakage.

Due to the multiplicity of semiconductor TiO_2_, there have many fabrication methods to decorate the TiO_2_ photoelectrode by changing TiO_2_ characteristics in order to enhance cell conversion efficiency [9, 10]. For instance, a mixture of TiO_2_ nanoparticles with different size, phase composition, and morphology result in conversion efficiency because of the light scattering and the facile electron transport [11, 12]. Alternatively, metal decoration of TiO_2_ photoanodes by tailoring photoanode properties, redistributing defects, and trapping levels in the band gap enables changing the conduction band position [13–15]. However, metal-decorated TiO_2_ suffers from thermal stability issue which is worse for electron-hole recombination. Oppositely, nitrogen and carbon doping on TiO_2_ can eliminate oxygen vacancies and surface-deficiency-related defects and results in lower resistivity and contact resistance on the carrier transport pathway.

In previous works, we concentrated on the mixture of core shell structures of TiO_2_ nanotube with nanoparticles and then presented the electron transport behavior related to DSSC conversion efficiency. Different shapes of conduct carrier transport behaviors in TiO_2_ photoanode were discussed. TiO_2_ phase is controlled at around 500°C by sintering process and the results demonstrate that sintering treatment can significantly affect crystal nanoporous TiO_2_ photoanode for DSSCs. In this study, we investigate the photovoltaic behavior using mixed-phase TiO_2_ structure as anode in DSSC cell. It is well known that TiO_2_ has three polymorphs phases in nature: rutile, anatase, and brookite; the band gap of rutile phase and anatase phase are 3.0 eV and 3.2 eV, respectively. By mixing the different contents of rutile phase into anatase matric and coating it on FTO-glass substrate to be photoanode, the cell employing the composited photoanode was investigated to find out the conversion efficiency of DSSC by material properties, carrier transport, and EIS measurement.

## 2. Materials and Methods

Prior to the fabrication of the TiO_2_ photoanodes, fluorine doped tin oxide (FTO) glass substrates were cleaned by the same volume ratio of acetone and isopropyl alcohol mixture in an ultrasonic water bath for 30 min. To prepare TiO_2_ nanoparticle paste, Degussa-P25 (anatase: 80%, rutile: 20%) powder and P90 (anatase: 90%, rutile: 10%) powder acting as TiO_2_ precursors were mixed with specific contents to meet experimental design. First, 1 g of polyethylene glycol (PEG) was hydrolyzed in 3 mL of deionized water under stirring at room temperature for 15 min. Second, separately P25 and P90 mixtures (w/w = 0%/100%, 30%/70%, 50%/50%, 70%/30%, 100%/0%) were add to PEG colloids, to obtain rutile weight percent from 5% to 15% which was denoted as R5, R9, R10, R14, and R15, respectively. Then, colloid was stirred at room temperature for 8 h. To prepare TiO_2_ photoanodes, first, spin coat FTO glass with TiCl_4_ and anneal substrate at 80°C for 50 minutes. Second, 10 um~14 um of synthesized mixed-phase TiO_2_ nanoparticle paste was formed on substrate using a glass rod. All as-prepared TiO_2_ photoanodes were calcined at 450°C for 30 min in air atmosphere to form microstructure.

An active area of 0.5 cm × 0.5 cm was selected from the TiO_2_ photoanode and immersed in a 3.0 × 10^−3^ M solution of the ruthenium based dye [RuL_2_(NCS)_2_] TBA_2_ for overnight, where Ru is ruthenium, L represents 2,2′-bipyridyl-4,4′-dicarboxylic acid, NCS stands for isothiocyanate, and TBA is tetrabutylammonium (N719 dye, Everlight Chemical, Taiwan). The specimens were washed with ethanol after immersing in N719 dye solution. A thin Pt sputtered on an FTO glass was used as the counterelectrode. The iodide/tri-iodide (I^−^/I_3_^−^) electrolyte (lodolyte R-150) was cast into the dye absorbed TiO_2_ electrodes. The TiO_2_ photoanode and the Pt coated cathode were clamped together in order to assemble the DSSC devices. The film morphology was observed by field-emission scanning electron microscope (FESEM). The crystalline phases of the obtained titanium electrodes were characterized by X-ray diffraction (XRD) radiation at scanning rate of 0.01 deg/min from 2*θ* = 5° to 60° and Raman spectroscopy from wavelength = 100 to 2000 cm^−1^. The photovoltaic characteristics of DSSC devices were measured by an electrochemical analyzer under a standard AM 1.5 sunlight illumination with 100 mW/cm^2^ light source. The electrical impedance spectra (EIS) were also measured in the range of 0.01 Hz to 100 kHz using the same equipment and setup. To obtain the information about band gap energy of the as-synthesized nanoparticle, spectrum has been recorded using UV/vis spectrophotometer. Photoluminescence (PL) spectroscopy was used to obtain the information about defects of the as-synthesized nanoparticle.

## 3. Results and Discussion

Characteristics of crystal TiO_2_ photoanode, resistance of carrier pathway, EIS curves, and photovoltaic results are overall discussed by the integrated DSSC cell.

### 3.1. Mixed TiO_2_ Photoanode Morphology and Properties

**Figure 1:**
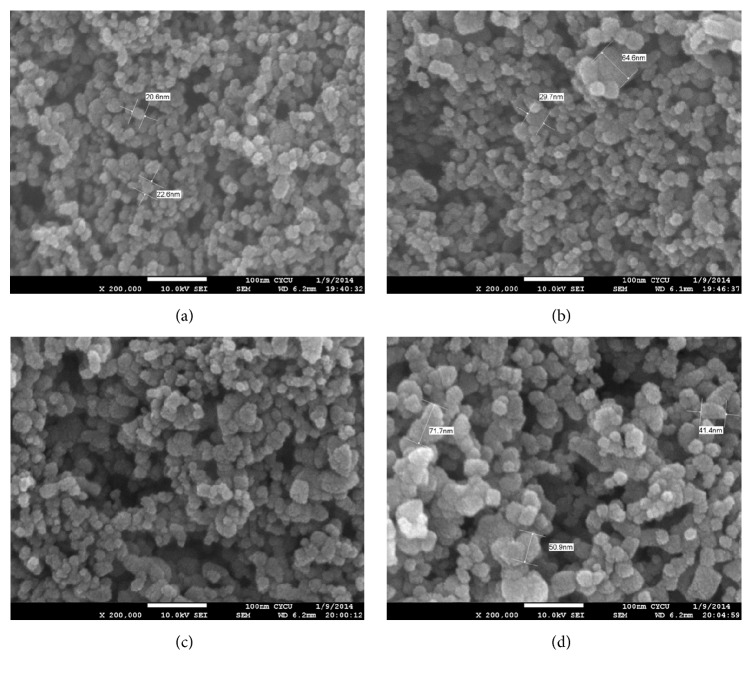
SEM images of the photoelectrodes top view: (a) R5, (b) R9, (c) R14, and (d) R15.

**Figure 2:**
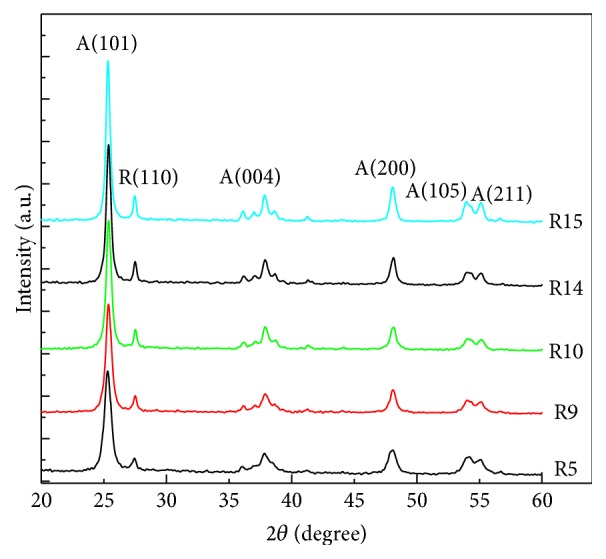
XRD patterns of TiO_2_ nanoparticles.

**Figure 3:**
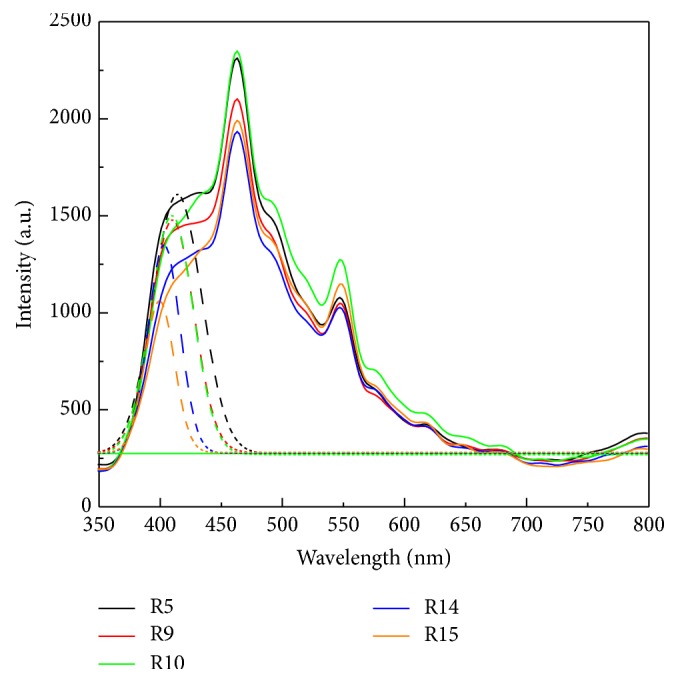
PL spectra of TiO_2_ specimens.

**Figure 4:**
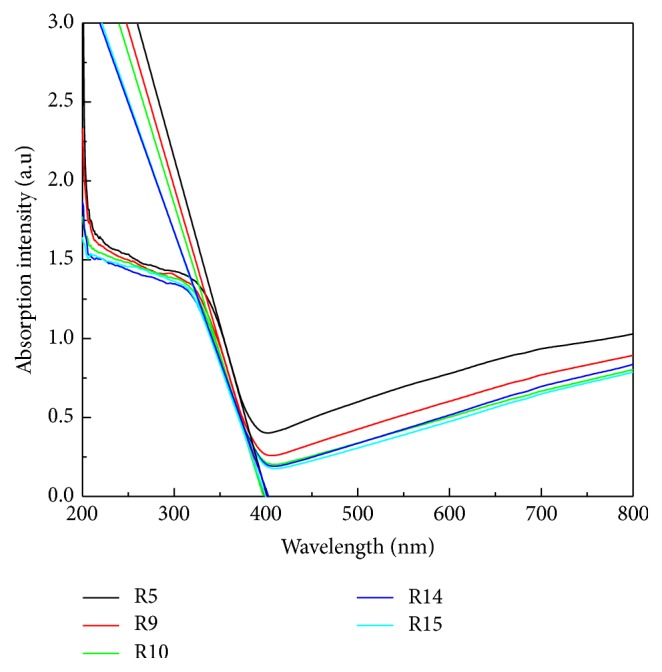
UV-vis spectra of TiO_2_ photoanodes.

To investigate morphology of various rutile contents and ensure good adhesive interface between mixed TiO_2_ nanocomposite and FTO before applying to DSSC, the mixture was used to be verified by SEM.  Figure 1 shows the top-view SEM images of TiO_2_ nanoparticles. It can be seen that TiO_2_ nanoparticles with approximate spherical shape has an average particle size around 20–30 nm of R5 and random size distribution from 30 nm to 70 nm in R9 to R15, respectively. The average particle aggregates as the rutile phase increases.  Figure 2 shows XRD diffraction patterns of synthesized TiO_2_ after sintering at 450°C which were used to investigate the crystallization degree, compositions, and grain size. Moreover, peak patterns can be used to estimate the anatase content in the nanostructured using the following equation:

(1) $$\begin{array}{c} C_{A}=\frac{A_{A}}{A_{A}+1.265\times A_{R}}\times 100\%, \end{array}$$

where *C*_A_ is the anatase content in the TiO_2_ and *A*_R_ and *A*_A_ are the areas covered by rutile peak (110) and anatase peak (101) in the XRD pattern, respectively. In Figure 2, R15 peak simultaneously shows anatase (101) and rutile (110) planes that imply the anatase and rutile phase are well-crystallized due to sharp diffractions. In contrast, R5 only has the sharp diffraction pattern associated with anatase (101) crystal plane and broad diffraction from the rutile (110) plane illustrates a relatively low degree of crystal structure of the rutile TiO_2_. Besides, the grain size was determined from the width at half maximum of the (101) anatase peak according to the Scherrer formula:

(2) $$\begin{array}{c} D=\frac{0.9\lambda }{w\times \cos\theta }, \end{array}$$

where *λ* is the X-ray wavelength, *w* is width at half maximum peak, and *θ* is peak position. Grain size is gradually agglomeration from 11.8 nm to 19.5 nm by increasing rutile content. Synthesized TiO_2_ nanocomposite was coated on fluoride-doped tin oxide (FTO) conducting glasses acting as photoanode.  Table 1 shows the results of different amount of the rutile phase and grain size in anatase matrix. The mixtures contain various ratios of rutile phase in anatase matrix which are denoted from R5 to R15, respectively, where the numbers represent the rutile content. Photoluminescence (PL) is a common method to analyze the characteristics of photogenerated charge trapping and carrier separation behavior, and the PL emissions result from the recombination of photogenerated charge carriers. In this work, the PL spectra excited by 325 nm light source was used to express the deficiency in TiO_2_ nanoparticles. As shown in Figure 3, three main signal peaks are found at 395 nm, 470 nm, and 545 nm. A peak centered at 395 nm is found among all samples which results from the transient of shallow donor level in forbidden gap. It is speculated that electron-trapped oxygen vacancy that formed shallow energy levels is located at forbidden gap and near conduct band, resulting in emission at near 395 nm wavelength [16]. These defect levels as well as surface states can act as chemisorptions of oxygen which control electron scavenge on the photoanode TiO_2_ surface [17]. Both R15 and R14 show lowest peak intensity at 395 nm, indicating lower electron-trapped oxygen vacancy of defect state level in band gap. Because the PL spectra of TiO_2_ are sensitive to the prepared conditions and crystal characteristic, sample R5 related to high lower crystalline could intrinsically chemosorb oxygen molecular and exhibit high intensity at peak 395 nm among all samples. The peak at 395 nm related to electron-trapped oxygen vacancy is the dominant factor to affect carrier transport in DSSC operation. A peak around 545 nm is attributed to the relaxation of self-trapped exciton which is thought to be electron-hole recombination rate in TiO_2_ structure. To be noted that this recombination within TiO_2_ photoanode is not major dominant rule in DSSC performance. A sharp peak at around 465 nm was found among all samples; the back-electron transfer at the interface of TiO_2_ photoanode electrolyte is illustrated to be the main recombination pathway in deteriorating the DSSC efficiency. Higher rutile content proposes lower defect state levels in band gap [18, 19]. UV-vis spectra were measured to investigate the effect of rutile content on the optical properties in anatase TiO_2_ matrix. Different rutile contents in anatase matrix are denoted as R5, R9, R10, R14, and R15, respectively.  Figure 4 indicates that the absorption edges of synthesized TiO_2_ materials were successfully extended to the visible region. All samples exhibit optical absorption below 400 nm which is attributed to the band-to-band electron transition in the TiO_2_ nanocrystals related to its band gap energy near 3.1 eV. It can be observed that the absorption thresholds at 400 nm for R5 samples are slightly blue-shifted compared to that of R15 one and the degree of blue shift slightly increases with the amount of rutile content. The integral band gap energy agrees with the full width at half maximum (FWHM) shown in Figure 2 XRD pattern, meaning the smaller FWHM has the better conductivity, and proposes narrow band gap energy [20, 21].

### 3.2. Photovoltaic Results of DSSC

**Figure 5:**
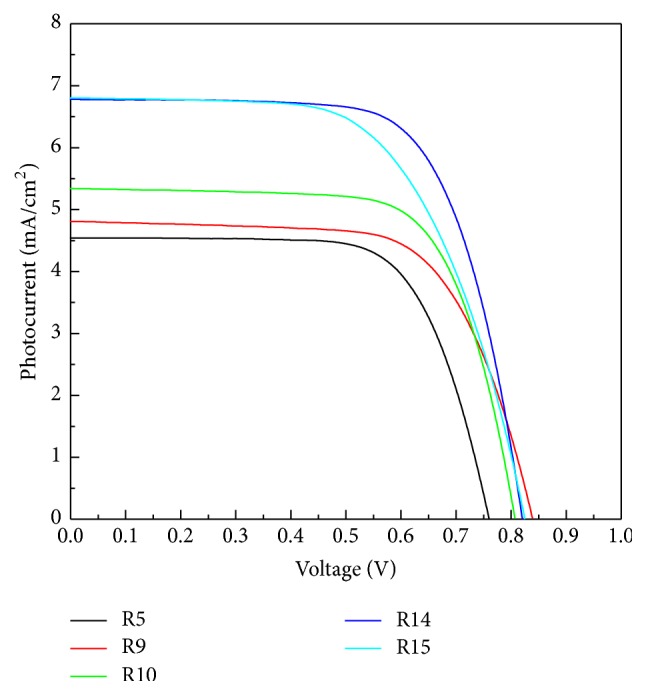
*J*-*V* plots measured by various TiO_2_ photoanodes of DSSC devices.

**Figure 6:**
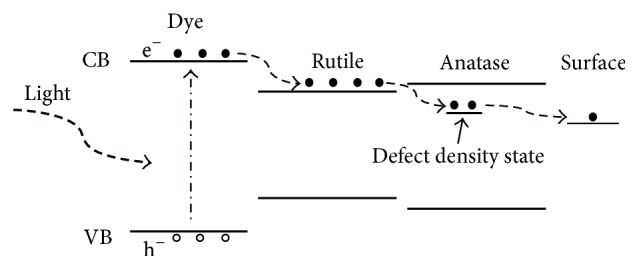
Carriers transport paths of anatase and rutile phases TiO_2_.

The photovoltaic characteristics of DSSC were measured under the intensity of 100 mW/cm^2^ simulated solar light. The overall solar conversion efficiency (*η*) is a product of the short-circuit current density (*I*_SC_), the open-circuit photo voltage (*V*_OC_), and the fill factor (FF), according to

(3) $$\begin{array}{c} \eta =\frac{I_{SC}\times V_{OC}\times FF}{P_{in}}, \end{array}$$

where *P*_in_ is the total light incident on the cell (100 mW/cm^2^).  Figure 5 shows the *J*-*V* curve; the values of the photovoltaic (PV) parameters including open-circuit voltage (*V*_OC_), photocurrent (*J*_SC_), fill factor (FF), and energy-conversion efficiency (*η*) are summarized in Table 2. The fill factor is the ratio of the maximum cell power to the product of *I*_SC_ and *V*_OC_. It can be seen from Table 2 that the highest overall efficiency (3.82%) was obtained at R14 and the cell performance was enhanced by rutile content (R14 > R15 > R10 > R9 > R5) until 14% of rutile content. Too much of rutile content is not good for energy-conversion efficiency. The synergistic effect between the anatase and rutile phases occurs in R14, suggesting that an optimal rutile percentage near 14 wt% obtains the best performance by mixed-phase DSSCs. It highlights the existence of a synergistic effect between the mixture TiO_2_ photoanode in DSSCs. It is known that efficiency of electron transfer is determined from the degree of recombination rates and pure rutile displays photocatalytic inactive due to high recombination center compared to anatase. By decorating rutile content in anatase matrix, slight rutile crystallizes and the defect state relating to electron-trapped oxygen vacancy and electron scavenge decreases. Therefore, the present rutile content prompts electron transfer from rutile to anatase lattice trapping sites, future inhibiting electron/hole recombination occurrence [22, 23].  Figure 6 shows the schematic drawing of the energy diagram, illustrating the pathway of excited photoelectrons injection from the dye to the rutile conduction band, passing through anatase trapped level in the band gap and arriving at photoanode surface [24].

### 3.3. Parameters of Electron Transport Determined by EIS

**Figure 7:**
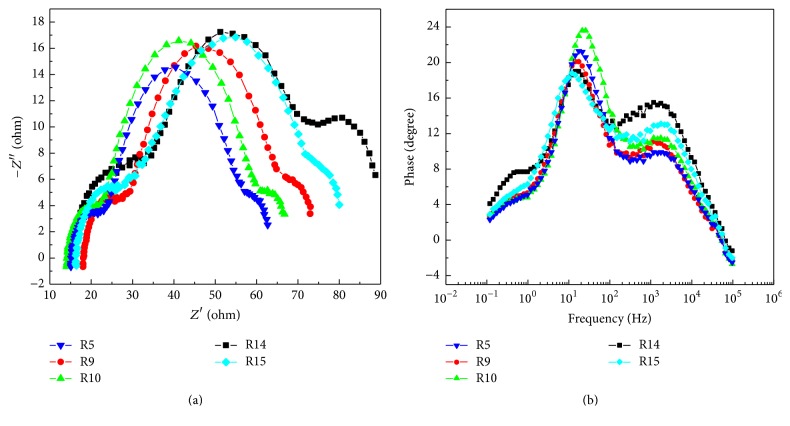
(a) Nyquist plots corresponding to specific resistance in photoanode/electrolyte/dye system. (b) Bode plots related to peak frequency.

There are three types of impedance and electron pathway in DSSC system including the recombination in TiO_2_-electrolyte dye and proposed carriers transport pathway. The total impedance (*Z*_*S*_) of the DSSC is given by the sum of the summation of impedance of diffusion and recombination in TiO_2_ photoanode (*Z*_*T*_), impedance at Pt electrode/electrolyte interface (*Z*_*P*_), and impedance of tri-iodide diffusion in the electrolyte (*Z*_*N*_). The impedance of *Z*_*T*_ consists of *R*_*W*_ and *R*_*K*_, which represents electron transport resistance in TiO_2_ photoanode and charge-transfer resistance related to electrons recombination with electrolyte, respectively. All electron transport parameters on DSSC including charge-transfer resistance (*R*_*k*_), electron density (*n*_*s*_), and electron life time (*τ*) on conduction band are evaluated by EIS measurement.  Figure 7(a) shows the typical experimental spectra of Nyquist plot; each sample contains three arcs (*ω*1, *ω*2, *ω*3) corresponding to charge transport within Pt counterelectrode and electrolyte interface, TiO_2_ photoanode-electrolyte-dye system, and diffusion of tri-iodide ions in the electrolyte, respectively [25]. The *R*_*k*_ value is estimated from the diameter of central arc *ω*2. Moreover, at the steady state, electron density *n*_*s*_ value in the conduction band is calculated as the following equations:

(4) Con=L×Keff×Rk,ns=KBTq2×A×Con.

Here *L*, *K*_eff_, *q*, *A*, *n*_*s*_, *K*_*B*_, and *T* represent photoanode TiO_2_ thickness, peak frequency of the central arc *ω*2, the charge of an electron, electrode area, electron density in the conduction band, Boltzmann constant, and absolute temperature. The life time (*τ*) of photoinjected electrons within TiO_2_ photoanode is calculated as 1/2*πf* and the highest peak frequency *f* is obtained via the Bode plot shown in Figure 7(b) whose frequency ranges from 1 to 100 Hz.  Table 3 summaries EIS parameters of all samples. Because higher *R*_*k*_ value could suppress the back-electron recombination at the TiO_2_ photoanode/electrolyte interface and then increases *V*_OC_, in contrast, surface state and oxygen vacancy acting as recombination pathway could simultaneously decrease *R*_*k*_. Although R5 is composed of high *n*_*s*_ value on conduction band and longer *τ*, lower *R*_*k*_ value 34.9 Ω deteriorates the efficiency of energy conversion due to lower *V*_OC_ of back-electron recombination. It consists with photoluminescence result that lower surface state and oxygen vacancies contained in high rutile content samples, >14% rutile content in anatase TiO_2_, obtain high electron life time, smooth migration, and density in CB; simultaneously the higher *R*_*k*_ facilitates the higher *V*_OC_ and *J*_SC_ values; thus the highest efficiency of energy conversion is achieved. Rutile content may affect relative location of rutile conduction band to anatase trapping site, resulting in increasing of electron density and efficiency of energy conversion.

## 4. Conclusion

The addition of rutile content in anatase TiO_2_ matrix prompts TiO_2_ crystalline and reduces the integral band gap and defect density states in forbidden gap. Moreover, light harvest on DSSC significantly enhanced the photocurrent and overall solar conversion efficiency by increasing rutile content into anatase photoanodes. The obvious increase in *J*_SC_ on the 14% rutile TiO_2_ phase in photoanode has the highest energy-conversion efficiency (*η*) of 3.8% and is 58% higher than that on 5%. The addition of rutile is the major reason to reduce oxygen vacancies and less electron-back recombination. In appropriate rutile content on anatase TiO_2_ matrix, the pathway of excited photoelectrons injection from the dye to the rutile conduction band, passing through anatase trapped level in the band gap and finally arriving at photoanode surface, becomes smooth. An optimal rutile content around 14 wt% is found to be increase the DSSCs performance with regard to photoanode crystalline, defect density level, and electron transport.

## Figures and Tables

**Table 1:** Value of the grain size and the rutile content of TiO_2_ photoanodes.

	Grain size (nm)	Rutile content
R5	11.78	5.29%
R9	14.89	8.97%
R10	16.47	10.34%
R14	18.12	13.89%
R15	19.46	15.04%

**Table 2:** Summary of photovoltaic parameters on DSSC under stimulated sunlight.

	*J*_SC_ (mA cm^−2^)	*V*_OC_ (V)	THK (um)	FF	*η* (%)
R5	4.544	0.761	9.6	0.7	2.4
R9	4.812	0.84	10.2	0.7	2.7
R10	5.34	0.807	12	0.7	3.01
R14	6.776	0.821	14	0.7	3.82
R15	6.812	0.825	10.5	0.6	3.42

**Table 3:** EIS measured results of various TiO_2_ photoanodes on DSSC devices.

	*R*_*k*_ (Ω)	*τ*_eff_ (ms)	*n*_*s*_ (cm^−3^)
R5	34.9	7.5	9.37*∗*10^17^
R9	39.47	9.13	6.70*∗*10^17^
R10	40.16	6.19	6.00*∗*10^17^
R14	42.18	11.08	1.53*∗*10^18^
R15	45.65	11.08	9.94*∗*10^17^
